# Inter-molecular β-sheet structure facilitates lung-targeting siRNA delivery

**DOI:** 10.1038/srep22731

**Published:** 2016-03-09

**Authors:** Jihan Zhou, Dong Li, Hao Wen, Shuquan Zheng, Cuicui Su, Fan Yi, Jue Wang, Zicai Liang, Tao Tang, Demin Zhou, Li-He Zhang, Dehai Liang, Quan Du

**Affiliations:** 1 Beijing National Laboratory for Molecular Sciences and the Key Laboratory of Polymer Chemistry and Physics of Ministry of Education, College of Chemistry and Molecular Engineering; State Key Laboratory of Natural and Biomimetic Drugs, School of Pharmaceutical Sciences; Institute of Molecular Medicine, Peking University, Beijing 100871, China; 2 Department of Obstetrics & Gynaecology, Faculty of Medicine, The Chinese University of Hong Kong, Shatin, New Territories, Hong Kong, China

## Abstract

Size-dependent passive targeting based on the characteristics of tissues is a basic mechanism of drug delivery. While the nanometer-sized particles are efficiently captured by the liver and spleen, the micron-sized particles are most likely entrapped within the lung owing to its unique capillary structure and physiological features. To exploit this property in lung-targeting siRNA delivery, we designed and studied a multi-domain peptide named K-β, which was able to form inter-molecular β-sheet structures. Results showed that K-β peptides and siRNAs formed stable complex particles of 60 nm when mixed together. A critical property of such particles was that, after being intravenously injected into mice, they further associated into loose and micron-sized aggregates, and thus effectively entrapped within the capillaries of the lung, leading to a passive accumulation and gene-silencing. The large size aggregates can dissociate or break down by the shear stress generated by blood flow, alleviating the pulmonary embolism. Besides the lung, siRNA enrichment and targeted gene silencing were also observed in the liver. This drug delivery strategy, together with the low toxicity, biodegradability, and programmability of peptide carriers, show great potentials *in vivo* applications.

Lung cancer and infection are common diseases worldwide^1^. In the past decades, nanoparticle-based lung-targeting drug delivery systems have been extensively investigated^2^,^3^. In which, pulmonary inhalation^4^,^5^,^6^ and intravenous injection are the two major routes of drug delivery. The airway-directed drug delivery systems have such advantages as low system exposure, high local drug concentration, and high tissue-specificity^7^. They are, however, not efficient strategies because of mucus clearance, particularly for the large or charged nanoparticles^8^,^9^. Different from inhalation, nanocarriers administrated intravenously are able to mediate selective accumulation in tumor region by means of enhanced permeability and retention (EPR) effects^10^,^11^. Moreover, ligand-attached nanocarriers may achieve active targeting by binding to the appropriate receptors^12^,^13^. Intravenous drug delivery has its own limitations. It has been reported that drug carriers with positively charged surface would preferentially accumulate in the liver, spleen, and kidney, leading to seriously dose-dependent cytotoxicity^14^.

Compared to the other tissues, lung is unique in its capillary structure and physiological functions, receiving all the venous return. Therefore, it has the priority to collect the substrates in the bloodstream before they are transported throughout the body^15^. Following intravenous administration, particles with sizes of 5 ~ 20 μm are effectively accumulated in the lung, due to the filtering effects of pulmonary capillary. This enables efficient and passive lung-targeting as reported^16^,^17^,^18^. Taking advantage of this unique property, several delivery systems have been designed for passive lung-targeting^19^,^20^,^21^. siRNA is a promising drug candidate in gene therapy^22^. Sun and co-workers^23^ reported that positively-charged siRNAs interacted strongly with serum to form aggregates about 5 μm in diameter, which were efficiently entrapped within lung capillaries. Following the same principle, Nakamura *et al*. achieved lung-specific siRNA delivery by using cationic tetra (piperazino) fullerene epoxide (TPFE)^24^.

Although larger particles facilitate lung-specific targeting, they also likely to cause pulmonary embolism if not removed or degraded in time. To this end, Anselmo *et al*. showed that under the shearing force of blood stream, nanoparticles absorbed on the surface of blood cell were able to be detached in capillary^25^. On the basis of this observations, we speculate that the siRNA complexes, if properly designed, are not only able to form large aggregate in serum and entrapped in the lung, but also subjected to shearing force generated by the blood flow and undergoing a gradual degradation, alleviating pulmonary embolism. Our previous study showed that the complexes formed by oligonucleotides and polyelectrolyte were smaller in size and exhibited a fast kinetics^26^. Incorporation of water molecules inside the complexes facilitated its formation^27^. Such complexes should have a strong and fast response to blood flow. Herein, we designed a short, multiple-domain peptide named k-β. It is composed of four functional blocks with different physiochemical properties (Fig. 1A). At its N-terminal, a cationic lysine block (KKKK)_5_ is designed to interact with negatively-charged siRNAs. A (VEVK)_5_ block composed of hydrophobic valine, anionic glutamate, and cationic lysine alternately is designed to function as a cross-linker between neighboring peptides by forming inter-molecular β-sheet structures. The β-sheet structure is used to stabilize siRNA/peptide nanoparticles. To optimize the formation of the β-sheet structure, a flexible spacer block (GGG) is inserted between (VEVK)_5_ and (KKKK)_5_ blocks. At the C-terminal, a neutral and hydrophilic block (NNN) is introduced to tune the surface hydrophobicity and charge density, which is crucial for the size and shear-responsiveness of the aggregates.

## Results

### K-β peptide and K-β/siRNA complexes

**Figure 1:**
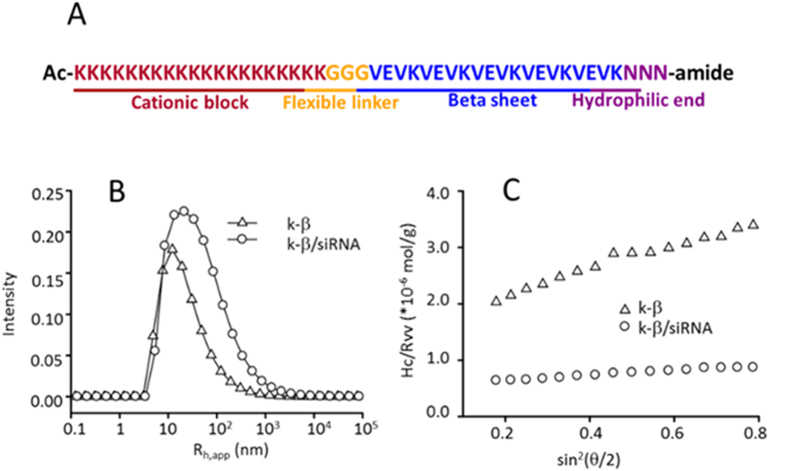
Characteristics of k-β and siRNA/k-β particles. (**A**) Sequence of k-β peptide; (**B**) Size distribution of k-β particles and siRNA/k-β particles in DPBS at 90° as determined by DLS; (**C**) Angular dependence of the scattering intensity of k-β and siRNA/k-β particles.

**Figure 2:**
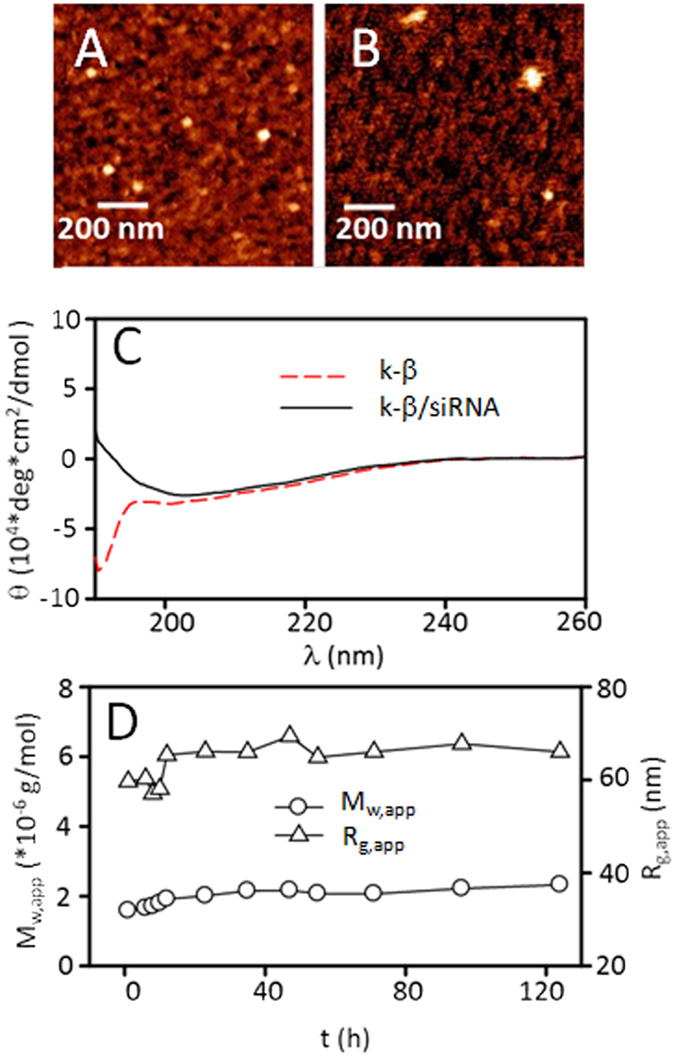
*In situ* liquid AFM images of k-β particles (**A**) and siRNA/k-β particles (**B**). (**C**) CD spectra of the particles; (**D**) Time dependence of *M*_w,app_ and *R*_g,app_ of the siRNA/k-β particles at pH 7.0. Concentration, 1.0 × 10^−4^ g/mL; N/P ratio, 20.

In DPBS solution, K-β peptides themselves form nanometer-sized particles. Laser light scattering (LLS) measurement reveals uniform particles with an apparent hydrodynamic radius (*R*_h,app_) of 20 nm at 0.1 mg/mL (Triangles, Fig. 1B). From the angular dependence of the excess scattered intensity, an apparent gyration radius (*R*_g,app_) of 39 nm and a weight-averaged molar mass (*M*_w,app_) of 6.0 × 10^5 ^g/mol are obtained for the particles (Triangles, Fig. 1C). This is further confirmed by *in situ* AFM imaging in liquid, showing spherical particles with a radius of 25 nm (Fig. 2A). Given a molecular mass of 5.0 × 10^3^ g/mol for the peptide and a contour length <6 nm, the complex particles shown in Fig. 1B are calculated to be composed of about 120 k-β molecules.

When siRNAs are added to k-β peptide solution, formation of siRNA/peptide nanoparticles occurs within a few minutes. For example, at a Nitrogen/Phosphate (N/P) ratio of 20, one diffusive mode with the hydrodynamic radius being ~30 nm is identified in LLS measurements (Circles, Fig. 1B), while spherical particles with a diameter of 60 nm are identified by liquid AFM imaging (Fig. 2B). Given d*n*/d*c* values of ~0.17 mL/g for both siRNA and peptide in solution, *M*_w,app_ and *R*_g,app_ of siRNA/peptide particles are calculated to be 1.8 × 10^6^ g/mol and 56 nm respectively, assuming that all the peptides and siRNA molecules complex completely (Circles, Fig. 1C). Both values are larger than those of the peptide-only particles. Given a molecular mass of 1.3 × 10^4^ g/mol of the siRNA, every complex nanoparticle is calculated to encapsulate ~56 siRNA molecules. Characteristics of siRNA/peptide particles at other N/P ratios are shown in Table 1 and Fig. S1A.

One crucial property of k-β peptide is that β-sheet structures are formed between neighboring molecules. To confirm this issue, circular dichroism (CD) spectral assays are performed with the peptide-only and the siRNA/peptide particles. While the assays reveal a random coil conformation for the peptide-only particles, β-sheet structures are identified for the siRNA/peptide particles as expected (Fig. 2C). Within these particles, β-sheet structures are speculated to serve as cross-linkers between neighboring peptide molecules, and further stabilized the overall particle structure. Supporting this speculation, particle stability is prolonged to 120 h (Fig. 2D, Fig. S1B).

According to the design, the negatively-charged glutamate residues are essential to the formation of the β-sheet structure, as well as the compact conformation of the particles. This hypothesis is tested by examining the formation of siRNA/peptide complexes at pH 3.0, at which conditions glutamate is not charged. The k-β peptide becomes a polycation at pH 3.0 rather than an amphiphilic molecule at pH 7.0. Under such condition, no β-sheet structure is formed (Fig. S2A). The siRNA/peptide complex has an *R*_h,app_ > 100 nm and a higher *M*_w,app_ (Fig. S2B,C). Without the formation of β-sheet structures, the intensity of the siRNA/peptide complexes is dropped dramatically by a factor of 6, likely due to the phase separation and precipitation (Fig. S2D).

To further characterize the stabilizing effect of the (VEVK)_5_ block, we designed two independent peptides without β-sheet-forming sequence, which are named (KKKK)_5_-*b*-(KGKG)_5_ and (KKKK)_5_-*b*-(KGGG)_5_ (Fig. S3A). CD spectra reveal a random coil conformation for these two peptides, with or without siRNAs. LLS measurements show that the excess scattered intensity of the complexes decreases right after a short increase at the very beginning, indicating a rapid increase in particle size (Fig. S3B,C). Consistently, extensive aggregation of the siRNA/peptide complexes occurs, resulting in a precipitation within a few minutes.

### K-beta/siRNA complex in serum under shear

**Figure 3:**
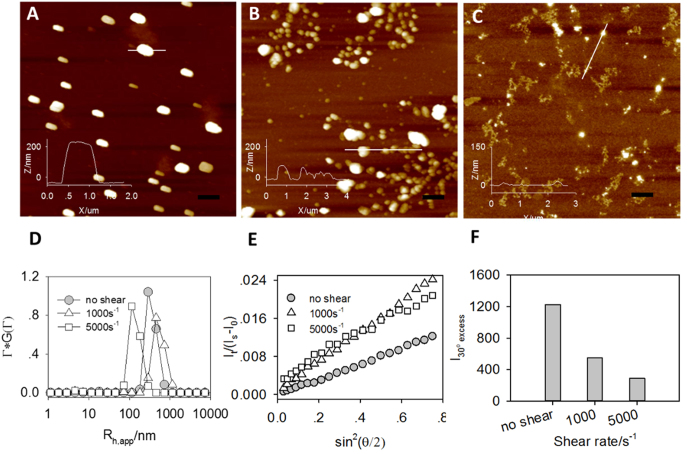
siRNA/k-β particles in the presence of 5% FBS under shearing. AFM images of the complex particles at a shear rate of 0 s^−1^ (**A**), 1000 s^−1^ (**B**), and 5000 s^−1^ (**C**) for 8 hrs, scale bar: 1 μm. The inset in each panel shows the height profile of selected particles. The bottom panels compare the size distribution (**D**), the angular dependence of the excess scattered intensity (**E**), and the excess scattered intensity at 30° (**F**) of the corresponding complex particles after being treated for 8 hrs.

To effectively accumulate in lung, k-β/siRNA complexes need to further associate into micron-sized aggregates in serum. Since human serum albumins are negatively charged under physiological conditions, they facilitate the aggregation with positively-charged k-β/siRNA complexes. Figure 3A shows the AFM image of the k-β/siRNA complex at N/P ratio = 20 in the presence of 5% serum. Aggregates with diameters of ~1 μm are formed in 8 hrs at static state. The height of the complex particle is more than 200 nm after dried. To avoid pulmonary embolism, the aggregates should be dissociated or degraded under physiological conditions. Depending on the dimension of blood vessels, the shear rate of human circulation is ranged from 1 to 10^5^ s^−1^^28^. Therefore, two shear rates (1000 s^−1^ and 5000 s^−1^) are chosen in our experiment. As shown in Fig. 3B,C, the sizes of the complex particle are much smaller after being sheared for 8 hrs. The corresponding heights of the particles are ~100 nm at 1000 s^−1^ and ~30 nm at 5000 s^−1^, indicating that a higher shearing force breaks down the complex particles into smaller sizes. LLS is also conducted to characterize the complex particles in aqueous solution after treatment of 8 hrs. Only one diffusive mode is observed in all the cases (Fig. 3D). However, the average size of the particle treated at 1000 s^−1^ is larger than that without shear, while the size of the particle treated at 5000 s^−1^ exhibits an opposite trend. The angular dependence of the excess scattered intensity (Fig. 3E), whose slope is proportional to the radius of the gyration of the particle, shows similar results. The excess scattered intensity, which denotes the molecular weight of the complex particles, is decreased with increasing shear rate (Fig. 3F). The chain density of a particle can be evaluated by *I*/*R*^3^. Calculation shows that the *I*/*R*^3^ value of the particle is dropped by about 6 times after being sheared at 1000 s^−1^ for 8 hrs, while the *I*/*R*^3^ value of the sample treated at 5000 s^−1^ is 3.5 times as high as that of the particle without shear, indicating that a higher shear rate could broke the aggregates into smaller but more compact particles, which is consistent with the previous report^29^. Together with the LLS and AFM data, we conclude that k-β/siRNA complexes form aggregates in the presence of serum. However, the aggregate is broken down into large and loose clusters under shear, which is able to further dissociate into smaller particles with increasing shear strength. Aggregation of k-β/siRNA complexes in serum and its responsiveness to shear rate are suitable for lung-targeting siRNA delivery. Note that the shearing force by blood flow only temporarily deforms or dissociates the aggregates. If the shearing force is removed, the particles with again form aggregates, even precipitates (Fig. S4).

### siRNA delivery in cultured cells

**Figure 4:**
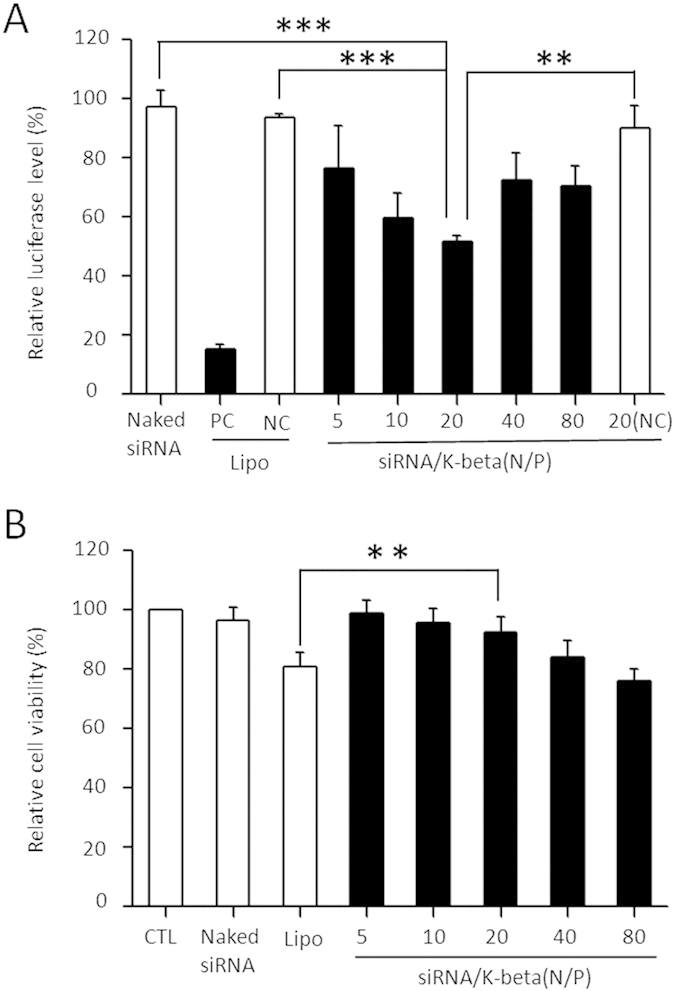
Gene-silencing potency and cytotoxicity in cultured cells. (**A**) Gene-silencing activities in cultured HEK293 cells. PC, effective siRNA targeting fusion luciferase gene; naked siRNA, luciferase-target siRNA without any delivery carrier; NC, negative control siRNA with an irrelevant target sequence with regard to the fusion luciferase gene; Lipo, transfection mediated by Lipofectamine 2000. (**B**) Relative cell viability after exposure to different siRNA/peptide formulations. CTL, untreated cells; naked siRNA, gene-specific siRNA without any carrier. Lipo, cells treated with Lipofectamine 2000. Concentration of siRNA is 33 nM. Only statistically significant associations are indicated. P-values are designated by asterisks (**P < 0.01; ***P < 0.001).

Gel retardation analysis suggests that the siRNA molecules bound to peptides are protected after the formation of complex. By means of a fusion reporter assay^30^, siRNA delivery potency of k-β peptide is examined with cultured HEK293 cells. Among the N/P ratios tested, the optimal gene-silencing activity is found at N/P = 20, leading to a 48% targeted gene repression (Fig. 4A). Using a lung carcinoma-derived epithelial cell line A549 and CCK-8 assay, no cytotoxicity is found with N/P ratios of 5, 10, and 20, compared to Lipofectamine transfection. When N/P ratios are further increased to over 20, evident cytotoxicity is observed (Fig. 4B).

To explore the contribution of the N/P ratio to gene silencing and cytotoxicity, comparative analyses are carried out in terms of particle size, aggregation, and the amount of siRNA. For N/P ratios >10, the *R*_h,app_ of siRNA/peptide particles is stable at ~30 nm, while the amounts of siRNA loaded decreases with increasing N/P ratio. At higher N/P ratios, the excessive positive charges lead to an increased cytotoxicity. For N/P ratios <10, an extensive aggregation occurs, resulting in irregular micron-sized particles. This compromises the uptake of the siRNAs and gene-silencing potency. Interestingly, different profiles are found for gene-silencing activity and cytotoxicity. This might reflect distinct mechanisms of the two processes.

### Lung-targeting siRNA delivery and gene silencing

**Figure 5:**
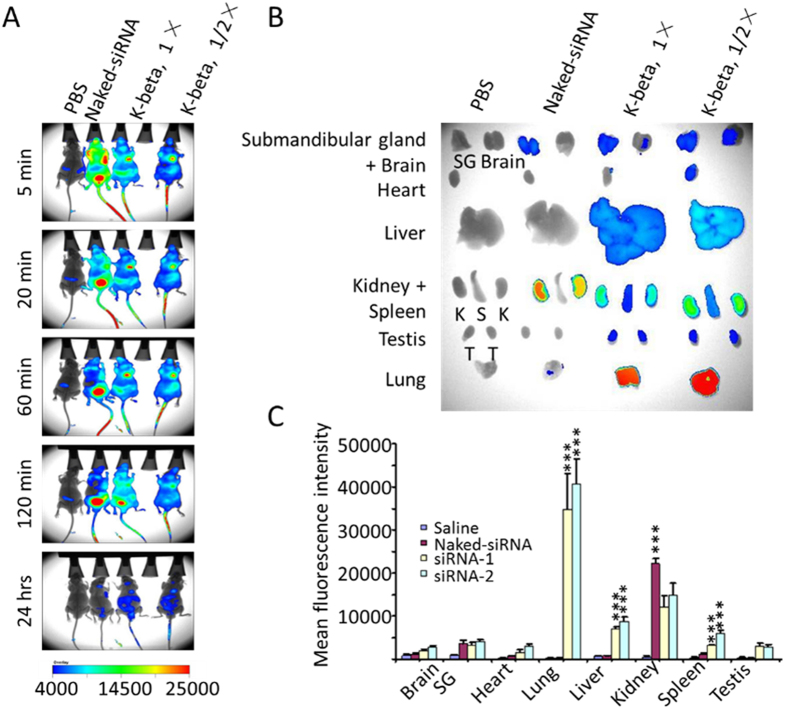
*In vivo* siRNA distribution. (**A**) Whole-body imaging. Three mice are included in each treatment group. (**B**,**C**) Twenty-four hours post administration, siRNA accumulation in the major tissues is visually examined (**C**) and quantified (**C**). Each bar represents a mean ± SEM of the siRNA levels accumulated in each tissue. PBS, mice treated only with saline; naked-siRNA, mice treated with gene-specific siRNA without any delivery carrier. Only statistically significant associations are indicated. P-values are designated by asterisks (***P < 0.001 versus corresponding naked siRNA control).

siRNA delivery is then performed in BALB/c mice, at N/P = 20 and a siRNA dose of 1.0 mg/kg. Fluorescence-labeled siRNA (25 μg), saline, or naked-siRNA is administered to each mouse *via* tail vein. Using an *in vivo* imaging system, the tissue distribution of the siRNAs is examined over a course of 48 h. Compared to naked-siRNA, the administration of peptide-formulated siRNA presents a significantly prolonged circulation time, and a distinct tissue distribution profile (Fig. 5A). As expected, the most intense siRNA signals are observed in the lung, followed by kidney, liver, and spleen (Fig. 5B,C). This particular tissue-specific distribution suggests that the size of the siRNA/peptide complexes is large in serum during circulation^24^, which supports our hypothesis.

**Figure 6:**
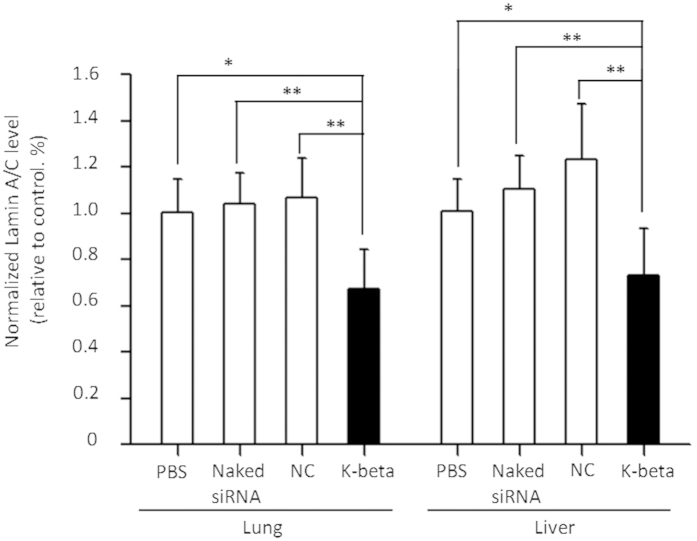
*In vivo* gene silencing 48 hours post siRNA administration. Repression of Lamin A/C gene expression by the siRNA formulation at an N/P ratio of 20, Six mice are included in each treatment group. PBS, mice treated only with saline; naked-siRNA, mice treated with gene-specific siRNA without any delivery carrier; NC, mice treated with a sequence-irrelevant control siRNA. Only statistically significant associations are indicated. P-values are designated by asterisks (*P < 0.05; **P < 0.01; ***P < 0.001).

To assess gene silencing *in vivo*, a Lamin A/C-targeting siRNA is delivered to mice, using k-β peptide formulation, naked-siRNA, or saline (Fig. 6). Compared to naked-siRNA or saline, treatment of siRNA/k-β formulation generates a targeted gene silencing of 30% in the lung and 27% in the liver, while no target gene silencing is observed in the kidney.

**Figure 7:**
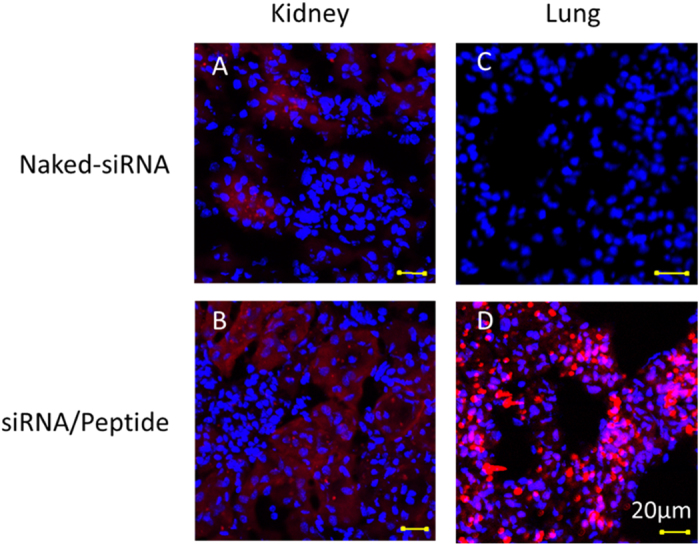
Tissue distribution of siRNA in lung and kidney 48 hours post siRNA administration. Hoechst 33342 counterstaining is used to indicate nuclei (blue) and siRNAs are labeled with Cy5 (red).

To further characterize siRNA delivery *in vivo*, tissue distribution of fluorescence-labeled siRNA is examined in the major tissues, using confocal microscopy (Fig. 7). Compared to naked-siRNA (Fig. 7C), peptide-formulated siRNA delivery shows greatly enhanced siRNA signals in the lung (Fig. 7D), in which uniform large particles of 2 μm are identified. It is in agreement with the LLS results as well as our hypothesis. Moreover, smear fluorescence periphery around the particle is also observed, suggesting a slow release of siRNA by itself or in k-β/siRNA complex form. In contrast to the lung, similar siRNA intensity and distribution for both naked-siRNA and peptide-formulated siRNA delivery are observed in the kidney (Fig. 7A,B).

In summary, these results indicate that after injection into the circulation, the nanometer-sized siRNA/peptide particles interact with serum proteins and undergo a second aggregation to form micron-sized aggregates. When these large aggregates pass through slow pulmonary circulation, they are entrapped within the capillaries, thus achieving passive accumulation, siRNA delivery, and gene silencing.

## Discussion

Even though tissue-specific delivery is the ultimate goal of siRNA therapy, most of the reported studies have been performed by means of passive accumulation of siRNA complexes within the liver, spleen, and kidney. This is mainly due to the differential property of the target tissues, as well as the physiochemical properties of the carrier. Virus infections, such as hepatitis virus infection, flavivirus infection, and Ebola virus infection, are common targets in siRNA therapy. Taking advantage of the high accessibility of liver, viral hepatitis is a promising target and also the mostly investigated disease in siRNA therapy. Lung infections are also very common and are notorious in causing considerable morbidity and mortality. For example, pneumonia is caused by bacteria, a virus, or fungi. While most people recover from pneumonia in one to three weeks, pneumonia can be life-threatening, particularly for those immunocompromised patients. Although it is possible to treat pneumonia with virus-specific siRNAs, these efforts present a formidable challenge, due to the lack of lung-targeting siRNA delivery reagent.

As a part of the circulatory system, lung is unique in its filtering capacity and entraps only large particles. This property excludes most of the current siRNA carriers, whose sizes are in the order of nanometers, from lung-targeting applications, because they are readily removed from the circulation by the liver and spleen. Taking advantage of the negatively-charged serum albumin and the pulmonary blood flow, we designed and studied a multiple-domain peptide carrier named k-β. By forming inter-molecular β-sheet structures when interacting with siRNAs, K-β peptides form stabile nanoparticles with siRNAs. A critical property of such particles is that when they are injected into mice, they further interact with serum to form micron-sized aggregates in the circulation. This enables them to be entrapped within lung capillaries, and lead to passive accumulation and gene-silencing in the lung. Such aggregates are deformed or dissociated under the shear caused by blood flow, alleviating the pulmonary embolism.

Besides siRNA delivery in the lung, siRNA enrichment and gene silencing is also observed in the liver. This observation may derive from the clearance of nanometer-sized siRNA/k-β complexes from the circulation by Kupffer cells or entrapping within hepatic sinusoids^31^. Although siRNA enrichment is further observed in the kidney, targeted gene silencing is not found. These results are in agreement with destiny of *in vivo* delivered siRNA, as free siRNAs or their degradation products are eliminated through renal filtration and end up in urine.

## Materials and Methods

### Materials

Peptide (purity >99%) was synthesized by GL Biochem. Ltd. (Shanghai, China). siRNA was from RiboBio Co., Ltd. (Guangzhou, China). Dulbecco’s phosphate-buffered saline (DPBS) was from Invitrogen (Shanghai, China). The sense strand sequence of the Lamin A/C-targeting siRNA was 5′-GCUACUGAACCACAAGAAUdTdT-3′, and the sense strand sequence of the irrelevant siRNA was 5′-GCCUGUUACUAUGAAAGAUdTdT-3′.

### CD spectra

CD spectrums of peptide-only and siRNA/peptide particles in DPBS buffer were measured from 190 to 260 nm using a JASCO J-810 spectrometer (AVIV, USA), with a 1.0 cm path length cuvette. For each sample, three independent assays were performed.

### Laser light-scattering (LLS)

For both static light scattering (SLS) and dynamic light scattering (DLS), a commercial spectrometer BI-200SM Goniometer (Brookhaven Instruments Corp.) was used, over a scattering angular range of 20–150°. A vertically polarized, 17 mW He–Ne laser operating at 633 nm (Research Electro Optics, Inc. Colorado, USA) was used as the light source. A BI-TurboCo Digital Correlator (Brookhaven Instruments Corp.) was used for data collection and processing.

In SLS, the angular dependence of the excess absolute time-averaged scattering intensity, known also as the Rayleigh ratio *R*_vv_(θ), was measured. In an extremely dilute solution, the weight-averaged molar mass (*M*_w_) and the root mean-square radius of gyration (*R*_g_) were calculated according to


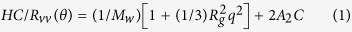


where *H* = 4π^2^*n*^2^(d*n*/d*c*)^2^/(*N*_A_*λ*^4^) and *q* = 4π*n*/*λ* sin(*θ*/2), with *N*_A_, *n*, d*n*/d*c*, and λ being Avogadro’s number, solvent refractive index, specific refractive index increment, and wavelength of the light in a vacuum.

In DLS, the intensity-intensity time correlation function *G*^*(2)*^*(τ)* in the self-beating mode was measured and calculated according to


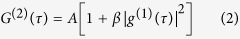


where *A* is the baseline in the measurement, *β* is the coherence factor, *τ* is the delay time, and *g *^*(1)*^*(τ)* is the normalized first-order electrical field time correlation function. *g* ^*(1)*^*(τ)* is related to the line width distribution *G*(Γ) as:


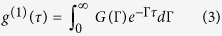


Using a Laplace inversion program, CONTIN, the normalized distribution function of the characteristic line width *G*(Γ) was calculated. The average line width, 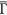, was calculated according to 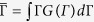. In which, 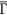 is a function of both *C* and *q*, which is expressed as:


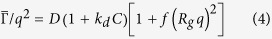


with *D, k*_*d*_, and f being the translational diffusive coefficient, the diffusion second virial coefficient, and a dimensionless constant. *D* was further used to calculate the hydrodynamic radius *R*_h_, according to the Stokes-Einstein equation


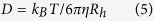


where *k*_*B*_, *T*, and *η* are the Boltzmann constant, and the absolute temperature and viscosity of the solvent.

In LLS measurements, the aqueous sample was first passed through a 0.20 μm syringe filter (Sartorius Stedim Biotech) to remove dust. siRNA/peptide complexes at different N/P ratios were prepared by adding siRNA to the peptide. N/P, the molar charge ratio, stands for the ratio of the positive charges of the peptides to the negative charges of the oligonucleotides.

### Atomic force microscopy (AFM)

AFM images were captured in tapping mode using a Multimode 8 (Brucker, Santa Barbara, CA). The Multimode 8 was equipped with a commercial 0.38 N/m constant force cantilever with an NP-S oxide-sharpened silicon nitride tip (Vecoo) and operated at a resonant frequency of ~9.4 kHz. 10 μL of the upper sample solution was deposited on a fresh mica surface using a pipette. After 30 seconds, the excess solution was blotted away with a strip of filter paper. The sample was air-dried for one day before AFM experiments. For liquid AFM, 30 μL sample was deposited onto the surface of a fresh mica; thirty seconds later, 100 μL DPBS was added into the chamber.

### siRNA complex under shear

MCR301 rheometer (Anton Paar, Graz, Austria) equipped with double-gap Couette geometry was used to apply the shear on the complex in serum. k-beta/siRNA complex at N/P = 20 was mixed with known amount of serum so that the final serum concentration in the complex solution was 5%. The mixed sample was loaded to the Couette and a shear at 1000 rad/s was then applied. The sample was taken out for measurement by LLS at chosen time point.

### Cell culture and RNAi assay

HEK293 cells were grown in Dulbecco’s modified Eagle’s medium (DMEM) supplemented with 10% fetal bovine serum, 100 units/ml penicillin, and 100 μg/ml streptomycin (Life Technologies). The cells were seeded into 24-well plates at ~1 × 10^5^ cells/well one day before transfection. A fusion reporter vector (0.17 μg/well) carrying the target site of the siRNA was transfected into HEK293 cells at ~50% confluence, together with a pRL-TK control vector (0.017 μg/well), with or without the siRNA of 33 nM. The activities of both luciferase reporters were determined by a Synergy HT fluorometer (BioTek, USA), then the firefly luciferase activity was normalized to Renilla luciferase for each well. The gene-silencing efficacy of the siRNA was calculated by comparison with cells without siRNA treatment. All the experiments were performed in triplicate and repeated at least twice.

### Cytotoxicity assay

To evaluate the cytotoxicity of k-β peptide, HEK293 cells were seeded in 96-well plates at ~6 × 10^3^ cells/well one day before transfection. After allowing them to grow overnight, the cells in each well were transfected with 0.08 μg siRNA using the k-β peptide carrier, or commercial Lipofectamine 2000 (Invitrogen). Four hours after transfection, 100 μL fresh complete DMEM was added to each well and the cells were further incubated for 20 h. Then, 20 μL of Cell Counting Kit-8 (CCK-8) reagent (Dojindo Molecor Technologies, Inc.) was added to each well and incubated for 2.5 h under normal conditions. At the end of incubation, the absorbance was read at 450 nm with a reference wavelength of 650 nm; the absolute absorbance (ODnet450) was calculated as the OD450 minus the OD650. For comparison of the relative viability, all data are presented as the mean percentage ± SEM, compared to the absorbance value of the mock-treated cells. Cell viability was calculated as:


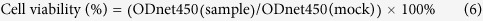


Where ODnet450(sample) is the absorbance of the transfected cells at 450 nm and ODnet450(control) is the absorbance of the mock control (non-transfected cells) at 450 nm.

### *In vivo* siRNA distribution and gene-silencing assay

All procedures used in animal studies were approved by the Animal Care and Use Committee of Peking University, and were consistent with local and state regulations as applicable. Male BALB/c-nu/nu mice 5–7 weeks old, weighing 18–22 g, were maintained in Peking University Laboratory Animal Center (an AAALAC-accredited experimental animal facility). A commercially available cy5-labelled siRNA was used in the biodistribution assay. A given formulation of the siRNA was administered to each mouse via tail vein injection at 1.0 mg/kg. The Cy5 fluorescence signal from the whole body was detected using an *in vivo* imaging system (Kodak FX Pro, Carestream Health), at given time points. At the end of the measurements, the mice were sacrificed by cervical dislocation. The major organs were isolated and examined using a Kodak *in vivo* imaging system. The organs were then cut into 6 μm sections and further examined using a confocal microscope.

To assess gene-silencing activity *in vivo*, a LaminA/C-targeting siRNA was injected into male C57BL/c mice. Mice 5–7 weeks old, weighing 18–23 g, were randomly divided into four groups and each given a single i.v. injection with a siRNA dose of 1.0 mg/kg, in formulation of k-β peptide/siRNA complex, naked siRNA, or siRNA-free phosphate-buffered saline (PBS). Forty-eight hours later, the mice were sacrificed and the lungs and liver were isolated for RNA extraction. cDNA was synthesized with SuperScript II (Invitrogen) and qPCR was performed using SYBR Green PCR mix (Invitrogen). Relative expression values were calculated (ΔΔ^CT^ method) using β-actin as an internal control. The PCR primers were as follows:

LaminA/C forward, 5′-GATGGAGGGCAATGTCAAGT;

LaminA/C reverse, 5′-AGAGGTGCAGATGGGAAATG;

β-actin forward, 5′-GAAGAGCTATGAGCTGCCTGA;

β-actin reverse, 5′-CTCATCGTACTCCTGCTTGCT;

sense sequence of the siRNA, 5′-CCAGCUAGAGCUGAGCAAAdTdT-3′;

antisense sequence of the siRNA, 5′-UUUGCUCAGCUCUAGCUGGdTdT-3′.

### Statistical analysis

Data were expressed as the mean ± SD or as the mean ± SEM, as indicated in the figure legends. Statistical variance was calculated by t-test, and P < 0.05 was considered statistically significant.

## Additional Information

**How to cite this article**: Zhou, J. *et al*. Inter-molecular β-sheet structure facilitates lung-targeting siRNA delivery. *Sci. Rep.* **6**, 22731; doi: 10.1038/srep22731 (2016).

## Supplementary Material

Supplementary Information

## Figures and Tables

**Table 1:** Size and siRNA loading of siRNA/peptide particles.

N/P	80	40	20	15	10	5
*R*_h,app_/nm	29	33	35	34	36	N/A
Number_(siRNA)_	37	50	57	67	87	N/A

N/A: No valid correlation curve was obtained due to the occurrence of macroscopic aggregation and precipitation.
