# Supplementary material for: Inter-molecular β-sheet structure facilitates lung-targeting siRNA delivery
Source: Sci Rep. 2016 Mar 9;6:22731. doi: 10.1038/srep22731 (PMC4783658; doi:10.1038/srep22731)
Supplement: Supplementary Information [file srep22731-s1.doc]

**Supporting Information for**

**Inter-molecular β-sheet structure facilitates lung-targeting siRNA delivery**

Jihan Zhou^1,+^, Dong Li^1,+^, Hao Wen^1^, Shuquan Zheng^1^, Cuicui Su^1^, Fan Yi^1^, Jue Wang^1^, Zicai Liang^1^, Tao Tang^2^, Demin Zhou^1^, Li-He Zhang^1^, Dehai Liang^1^*, Quan Du^1^*

^a^Beijing National Laboratory for Molecular Sciences and the Key Laboratory of Polymer Chemistry and Physics of Ministry of Education, College of Chemistry and Molecular Engineering; State Key Laboratory of Natural and Biomimetic Drugs, School of Pharmaceutical Sciences; Institute of Molecular Medicine, Peking University, Beijing 100871, China.

^b^Department of Obstetrics & Gynaecology, Faculty of Medicine, The Chinese University of Hong Kong, Shatin, New Territories, Hong Kong, China.

**Figure S1**. (**A**) Size distribution of siRNA/k-beta particles at various N/P ratios; (**B**)Time dependence of the size distribution at N/P = 20. Concentration of k-beta peptide, 1.0 × 10^-4^ g/mL; concentration of siRNA, 1.0 × 10^-5^ g/mL.

**Figure S2.** Complex formation of k-beta peptides and siRNAs at pH 3.0.


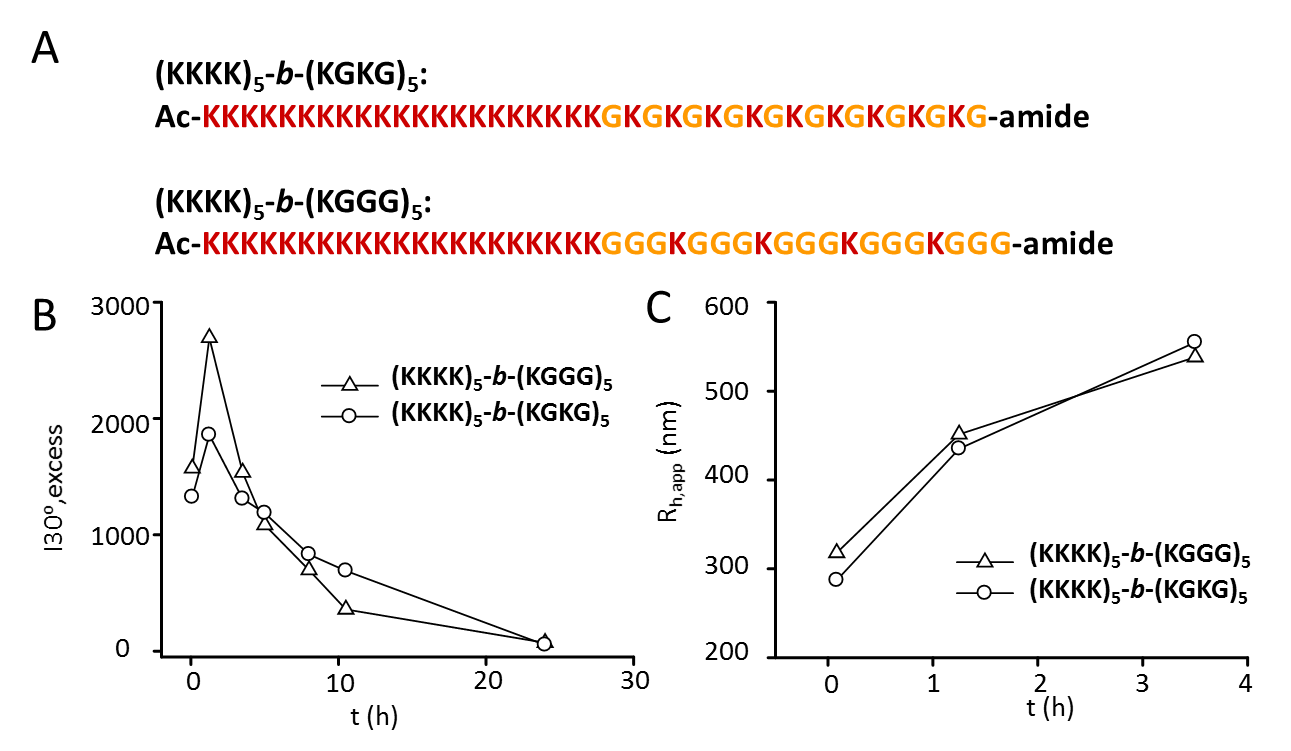


**Figure S3**. Complex formation of siRNA with two blocked peptides without the β-structure-forming sequence. (**A**) Sequences of blocked cationic peptides; (**B**) Time dependence of the excess scattered excess intensity of (KKKK)_5_-b-(KGKG)_5_/siRNA or (KKKK)_5_-b-(KGGG)_5_/siRNA complexes at 30°; (**C**) Time dependence of R_h,app_ of (KKKK)_5_-b-(KGKG)_5_/siRNA or (KKKK)_5_-b-(KGGG)_5_/siRNA complexes; No R_h,app_ was obtainable after 4 h since no valid correlation curves were collected due to the extreme fluctuation of the intensity.


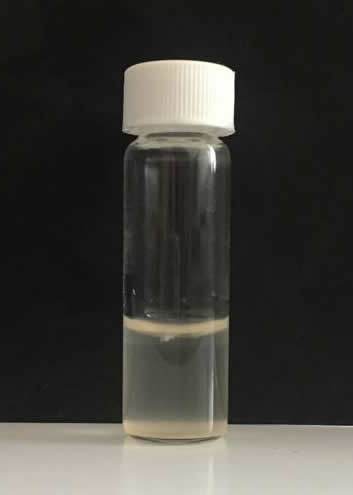


**Figure S4**. Picture of siRNA/k-beta complexes in the presence of 5% serum. The sample is sheared at 5000 s^-1^ for 8 hrs, and then left at room temperature for 12 hrs. The sample becomes cloudy and forms precipitate on the bottom of the vial.
